# New polymorphisms of *Vkork1* gene related to anticoagulant resistance of rats and mice in Italy

**DOI:** 10.1002/ps.8652

**Published:** 2025-01-15

**Authors:** Alessandro Reggiani, Gianluca Rugna, Eugenia Polverini, Rodolfo Veronesi, Romeo Bellini, Claudio Venturelli, Filippo Maria Dini, Roberta Galuppi, Guglielmo Pampiglione, Michele Dottori, Elena Carra

**Affiliations:** ^1^ Experimental Zooprophylactic Institute of Lombardy and Emilia‐Romagna Brescia Italy; ^2^ Department of Mathematical, Physical and Computer Science University of Parma Parma Italy; ^3^ Agriculture Environment Center “G. Nicoli” Crevalcore Italy; ^4^ Azienda Unità Sanitaria Locale della Romagna Ravenna Italy; ^5^ Department of Veterinary Medical Sciences University of Bologna Bologna Italy; ^6^ Pest Management Consultant Forlì Italy

**Keywords:** pest control, anticoagulant rodenticide, resistance, *Vkorc1*, molecular biology, molecular docking

## Abstract

**BACKGROUND:**

Anticoagulant rodenticides (ARs) are a very effective tool to control rodent pest populations. Nevertheless, AR resistance has been documented worldwide.

ARs block the cycle of vitamin K, leading to the death of the animal by internal bleeding: mutations in *Vkorc1* gene can cause resistance.

The spreading of AR‐resistant rodents could lead to an increase of their populations, the associated diffusion of zoonotic pathogens, and to the amplified exposure of non‐target animals to ARs, thus it is important to study its diffusion widely. This study aimed to report firstly the presence of *Vkorc1* mutations in synanthropic rodents from the Emilia‐Romagna region, Italy, and evaluate their role in resistance by means of molecular docking analysis.

**RESULTS:**

A total of 67 animals were analyzed: 24 *Rattus norvegicus*, 35 *Rattus rattus* and eight *Mus musculus*. Single nucleotide polymorphisms (SNPs) associated with AR resistance, in homozygosis or heterozygosis, were detected in 6/8 mice, on codons 128 and 139, and 13/24 *R. norvegicus*, on codons 61 and 139, and in 10/35 *R. rattus*, on codon 59. Furthermore, several newly described missense mutations were detected in all the tested species: the molecular docking analysis suggests a role of some of these (e.g., I123S and F87L) in resistance to brodifacoum, both in rats and in mice.

**CONCLUSION:**

The discovery of AR resistance SNPs in the 43.28% of tested rodents sounds as an alarm bell that requires the introduction of an integrated control approach, where ARs are not used routinely, but under specific monitoring evaluation. © 2025 The Author(s). *Pest Management Science* published by John Wiley & Sons Ltd on behalf of Society of Chemical Industry.

## Graphical abstract

This study firstly reports on *Vkorc1* gene mutations associated to anticoagulant rodenticide (AR) resistance in rats from Italy, and in rodents from Emilia‐Romagna region. New mutations probably involved in resistance have been described and the impact of these on rodenticide resistance was then studied via molecular docking with AR.

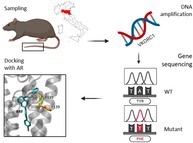

## 1 INTRODUCTION

Rodent pests are responsible of a variety of negative effects on human activities and public health,^1^ the main role being exerted by synanthropic species such as the brown or Norway rat (*Rattus norvegicus*, Berkenhout, 1769), the black rat (*Rattus rattus*, Linnaeus, 1758) and the house mouse (*Mus musculus*, Linnaeus, 1758). These animals may gain access to stored products, thus causing economic damage, as well as be reservoir or vector of several pathogens.^2^, ^3^ For this reason, beginning from the 1940s, anticoagulant rodenticides (ARs) have been used to control the populations of the pests^4^: these molecules are mostly coumarine derivatives, and exert their effect by inhibiting vitamin K epoxide reductase (VKOR) multiprotein enzymatic complex, competing with vitamin K for the same binding site, thus blocking the vitamin K cycle.^5^ ARs have become widely used in time, due to their property of causing a late death which prevents the development of avoidance behaviour in the rodent population, the versatility of administration forms (baits, powders, tablets) and cheapness if compared to mechanical traps.^6^

The insurgence of resistance to ARs was initially noticed in the 1950s in the UK,^2^, ^7^ and later in several other countries in Europe^7^, ^8^, ^9^, ^10^ and throughout the world.^11^, ^12^, ^13^, ^14^ In Italy, just a few studies have been conducted to assess the presence of resistance in the rodent population.^15^, ^16^, ^17^, ^18^

Anticoagulant resistance has a potentially serious impact on the efficacy of rodent control campaigns both in the public and private sectors, targeting the three main commensal and synanthropic species, such as *R. norvegicus*, *R. rattus* and *M. musculus*, widely spread throughout the Italian peninsula including the islands.^19^

Resistance to ARs is mainly linked to single nucleotide polymorphisms (SNPs) found in the three exons of the *Vkorc1* gene,^5^, ^20^ encoding the main subunit of the VKOR complex, which induce amino acidic substitutions conferring resistance to ARs by preventing a proper binding of the anticoagulant to the enzyme.^20^ Some of these SNPs of the *Vkorc1* gene, like at codons 128 and 139, represent ‘hotspots’ for mutations, as they were identified in rats and in mice.^20^

Following the evidence of resistance, new molecules have been developed to overcome the constraint, leading to the production of a ‘second generation’ of anticoagulant rodenticides (SGARs). These are quite similar to the ‘first generation’ (FGARs), but present larger side chains conferring greater efficacy.^2^ Unfortunately, also resistance to SGARs has been increasingly observed.^2^ In addition, the use of chemical controls may lead to unwanted side effects: ARs may disperse into the environment directly or be consumed by non‐target animals, then climb the food chain if predators eat poisoned animals. Many studies have reported the secondary or tertiary exposure of mammal carnivores to ARs throughout the world, particularly in the Northern hemisphere^21^: focusing on our region, in the period 2018–2022 wolves found dead in northern and central Italy were analyzed for the presence of ARs in their livers, with 115/186 (61.8%) of them testing positive,^22^ showing that dissemination of ARs is a wide‐spread problem. This adds up to the numerous reports of birds of prey and scavenger birds that have been found positive to ARs, particularly SGARs, in their livers and, sometimes, with evidences of AR poisoning.^23^, ^24^, ^25^

The effects on non‐targets due to the lack of selective toxicity together with persistence and bioaccumulation as well as the problem of resistance are the main topics that the current European and Italian legislation take into consideration with the aim of regulating the production, marketing and use of ARs.

The prohibition of ‘Permanent Baiting’ of ARs has therefore been introduced with the obligation of administration through tamper‐evident bait stations, in quantity and only for the time necessary to reduce the population of the rodent, where any other preventive technique may result as inappropriate.^26^, ^27^

Residues of VKOR enzyme whose mutation confer strong resistance to anticoagulants, are most likely located at the binding pocket, but they can be found also distributed in the cap domain, a region that covers the binding pocket and is stabilized/stabilizes ARs binding. In fact, two mechanisms were suggested to be related to this resistance: (i) the mutation directly affects the ligand binding; (ii) the mutation destabilizes the cap domain.^28^ The former mechanism is suitable for a docking simulation approach, a computational technique that tries to predict the most favorable ligand binding mode – therefore the ligand–receptor complex structure – knowing the molecular structure of the receptor and of the ligand itself. At the same time, an evaluation of the binding energy of the ligand in the best complex is performed. Here, we tried to use docking simulations to investigate the changes in the binding affinity due to the variation of the ligand environment caused by the mutation of selected residues. The higher the value the binding energy assumes, the more unfavorable the bond is. In the case of VKOR, this can reflect a resistance to ARs.

Purpose of this study was, therefore, to identify SNPs in the *Vkorc1* gene of rodent populations from the Emilia‐Romagna region (RER), in Italy, with particular attention to those altering the protein sequence. Then, the mutations experimentally found in mouse and rat that could potentially be related to anticoagulants resistance, were analyzed and selected for molecular docking simulations, on the basis of their proximity to the binding site.

## 2 MATERIALS AND METHODS

### 2.1 Sample collection

**Figure 1:**
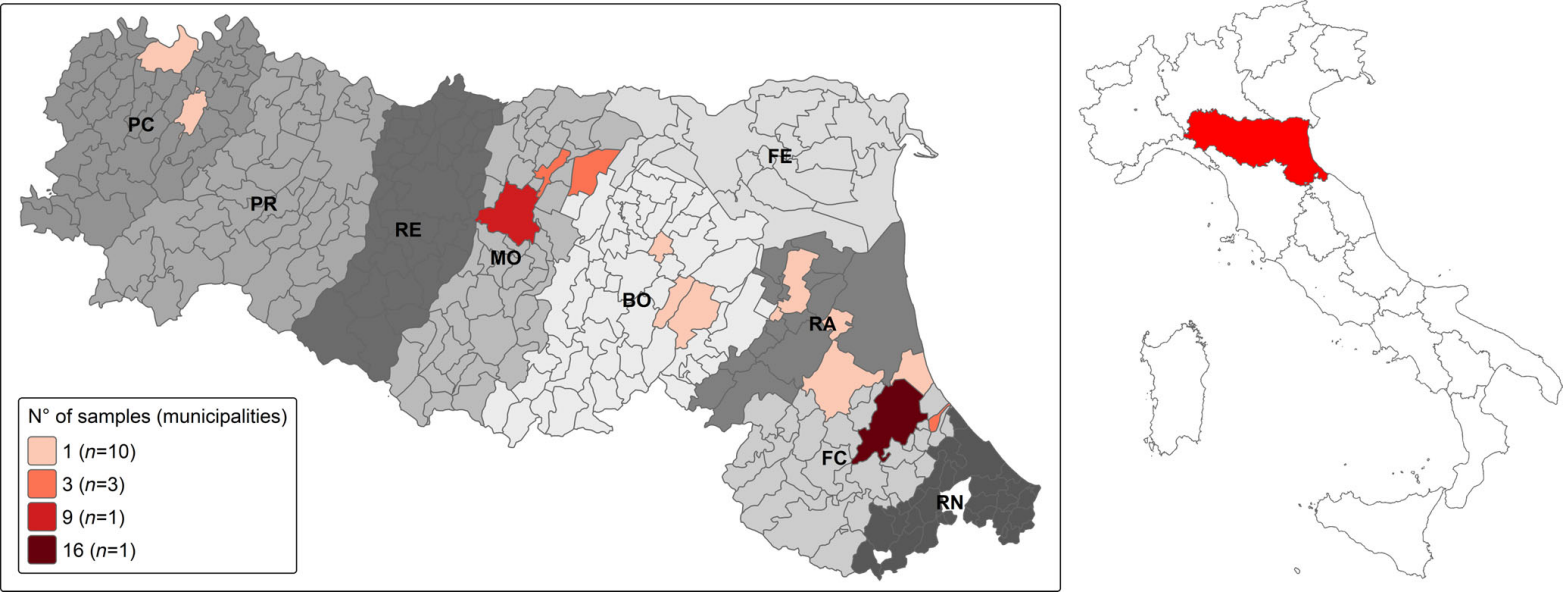
Map of Emilia‐Romagna region (RER), with its location depicted in red in the map of Italy. The different shades of grey indicate the nine provinces within RER: Piacenza (PC), Parma (PR), Reggio Emilia (RE), Modena (MO), Bologna (BO), Ferrara (FE), Ravenna (RA), Forlì‐Cesena (FC), Rimini (RN). Municipalities where rodents were sampled are highlighted in a shade of red proportionally darker according to the number of samples. Please note that 23 rodents, not shown on the map, where captured in the provinces of FC and RA but the exact location is not known due to privacy reasons.

Samples were collected during 2023 thanks to the collaboration of some pest management companies, operating in RER, that were asked to confer carcasses or tails of rodents to our institution (IZSLER), instead of destroying them according to legislation (Regulation EC No. 1069/2009). Sampling strategy was approved by the Ethics Committee of IZSLER (protocol number: 16194/2023). A total of 67 rodents were collected, from different provinces of RER (Fig. 1). The cause of death was known to be bromadiolone (SGARs) just for four black rats and one brown rat, while for the other specimens it was either predation or traps or, in most cases, not known.

Carcasses underwent a necropsy analysis, during which the tail was collected for subsequent DNA extraction, while other organs were sampled for routine public health analyses. Tails were kept at −80 °C or in 80% ethanol until DNA extraction.

### 2.2 Sequence analysis

#### 2.2.1 DNA extraction and quantification

Tail muscle (25–30 mg) was added with 400 μL of ATL buffer (Qiagen, Hilden, Germany) and 40 μL of proteinase K (Qiagen), vortexed and put overnight at 56 °C while shaking. DNA was then extracted on a QIAsymphony (Qiagen) automated nucleic acid extractor, using QIAsymphony DSP Virus/Pathogen kit (Qiagen), according to the manufacturer's instructions, and eluted in a final volume of 60 μL. DNA eluate was then quantified by ultraviolet (UV) absorbance at 260 nm and 280 nm using a Synergy HTX multimode reader (Biotek–Agilent, Santa Clara, CA, USA), according to the manufacturer's instructions, then diluted with nuclease‐free water in order to have a final concentration in the range of 25–50 ng/μL.

#### 2.2.2 Species identification

Species information of each animal was initially determined morphologically during necropsy. However, to obtain a certain identification, mithocondrial cytochrome oxidase subunit I(COI) gene sequencing was performed using published protocols.^11^, ^29^ Polymerase chain reaction (PCR) amplicons were purified using the Agentcourt AMPureXP PCR Purification Kit (Beckman Coulter, Indianapolis, IN, USA) and checked via agarose gel electrophoresis. Sanger sequencing reactions were carried out in both forward and reverse directions using the GenomeLab™ DTCS Quick Start Kit (Beckman Coulter), in a final volume of 20 μL according to the manufacturer's instructions. Sequencing primers were based on M13 sequences, as described in the literature.^11^, ^29^ Sequencing reactions were purified using the Agentcourt CleanSEQ kit (Beckman Coulter), then Sanger sequencing was performed on a Sciex GenomeLab™ GeXP sequencer (Beckman Coulter). Sequences obtained were checked by a BLAST (Basic Local Alignment Search Tool) search in GenBank (NCBI) and in BOLD (Barcode Of Life Data), to confirm the correct species, based on the percentage of identity with available sequences. COI sequences from our specimens were deposited on the GenBank database (A.N.: PQ199645–PQ199711).

#### 2.2.3 *Vkorc1* gene amplification and sequencing

The regions including the three exons of *Vkorc1* gene were amplified following protocols already published in the literature, lightly modified to match the requirements of the GoTaq G2 Hotstart colorless Master Mix (Promega, Madison, WI, USA) that we used. PCR conditions were a bit different between mice and rats, but the same for all three exons of the same species, with the exception of the annealing temperature that was primer pair‐specific (Table 1). For mice: initial denaturation at 95 °C for 2 min; 35 cycles of denaturation at 94 °C for 45 s, annealing for 30 s at specific temperature, extension at 72 °C for 50 s; final extension at 72 °C for 5 min. For rats: initial denaturation at 95 °C for 2 min; 35 cycles of denaturation at 94 °C for 30 s, annealing for 30 s at specific temperature, extension at 72 °C for 30 s; final extension at 72 °C for 5 min.

**Table 1:** Primers used for amplifications and sequencing of *Vkorc1* exons

Species	Exon	Primer set	Ta (°C)	Length (bp)	Reference
*Mus musculus*	1	VKORC1_ex1_F‐TCTTCCCTCCTGTSYCTGGG	52	253	Iannucci *et al*.^16^
		VKORC1_ex1_R‐AAATYATCTGGYAACCTGGC			
	2	VKORC1_ex2_F‐CTGTGCTGAGGGGACAAAGT	50	801	
		VKORC1_ex2_R‐TTGCCATAAAACTGAGATTGTGA			
	3	VKORC1_ex3_F‐TTTCACCAGAAGCACCTGCTGYC	54	308	
		VKORC1_ex3_R‐ACACTTGGGCAAGGSTCATGTG			
*Rattus* spp.	1	RE1AF‐CTCTTGTGTCTGCGCTGTAC	60	~250	Mooney *et al*.^8^
		RE1R‐GCTTTTCATTTCTGCACGCA			
	2	RE2CF‐GGGTGGCGCTTCTTGCTAA	60	~350	
		RE2BR‐ACTCCTGCTAAGTGTTCTCCTTG			
	3	Rn_ex3_F‐CATTGGGGAGGTGTTACAGAG	58	~300	Iacucci *et al*.^15^
		Rn_ex3_R‐GATACACTTGGGCAAGGCTC			

*Note*: annealing temperature (Ta), amplicon length and reference are reported.

Primers, annealing temperatures, amplicon lengths and references are detailed in Table 1.

Amplicon and sequence purifications were performed as described earlier.

For *Vkorc1* gene sequencing, the primers used were the same of the amplification (Table 1), according to the protocol described earlier.

#### 2.2.4 *Vkorc1* sequence analysis

From each amplicon sequence, the exon region was obtained and exported in three multiple sequence Fasta files (one per exon), being very careful to have the same sample order in each file. An R tool, available at https://github.com/allereggiani22/Anticoagulanti/tree/main under a BSD 2‐Clause license, was then developed to join the sequences of the three exons to obtain the complete coding sequence (CDS) of each animal. These were then aligned and compared to a reference sequence retrieved from GenBank (A.N. AY423047 for *Rattus* spp. and A.N. NM178600 for *M. musculus*), using Bioedit Sequence alignment Editor (version 7.0.5.3),^30^ to identify all SNPs, regardless of their impact on the protein sequence. We decided to use the same *R. norvegicus* reference (A.N.: AY423047) for both rat species,^11^ because the two aminoacidic sequences differ just for the I90L mutation (as confirmed by *R. rattus* reference A.N.: XM032892517.1) and using the same reference could highlight species‐related SNPs due to a different codon usage. The complete CDSs obtained from our specimens were deposited on the GenBank database (A.N.: PQ204368–PQ204434).

### 2.3 Molecular docking simulations

#### 2.3.1 Brodifacoum ligand

Brodifacoum was chosen as a paradigmatic SGAR suitable to test the resistance effects and to compare the difference in binding energy due only to the inserted mutation and not to the use of a different ligand. In fact, from one side brodifacoum possesses – as well as the other SGARs – a large side group that occupies the entire binding pocket, increasing the binding area and resulting in strong inhibition of the wild‐type (WT) protein activity^28^; from the other side, the availability of the resolved crystal structure of the complex of brodifacoum with human VKOR^28^ in the PDB databank,^31^ makes it useful for structural comparison and validation of the docking results. The atomic coordinates of brodifacoum were obtained from such a complex (PDB ID 6wvh). Both hydrogen atoms and the Gasteiger partial atomic charges^32^ were added to the ligand by means of the Chimera 1.17.3 software.^33^ Brodifacoum was subjected to a light energy minimization to optimize the geometry of the structure, by means of the same software, using Amber ff14SB force field. The resulting conformation was used for docking simulations, keeping all the rotatable torsions of the structure free to rotate.

#### 2.3.2 VKOR protein

VKOR complex is an endoplasmic membrane enzyme involved in blood coagulation through the vitamin K cycle.^34^ The molecular structure of VKOR is characterized by a transmembrane four‐helix bundle that hosts the vitamin K binding site. The latter faces a large and flexible endoplasmic reticulum (ER)‐luminal region, which contains four catalytic cysteines. Such region includes, among other structural elements, a cap domain that covers the binding pocket.^28^ Both vitamin K and its antagonists that act as anticoagulants bind in the large and hydrophobic binding pocket, that also includes a tunnel formed between two of the transmembrane helices.

The sequence alignment of human, house mouse, brown and black rat VKOR, was performed with Clustal Omega 1.2.4 web server.^35^ The alignment showed an 81.4% of identical residues, reaching 93.8% if also the conservative ones are counted (Supporting Information Fig. S1).

The atomic coordinates of the WT structures of VKOR of house mouse, black and brown rat were downloaded by the AlphaFold database^36^ by means of the Uniprot sequence database interface,^37^ referring to the sequence codes Q9CRC0 (*M. musculus*), Q6TEK4 (*R. norvegicus*) and I4DXG3 (*R. rattus*).

The comparison of the rat and mouse structures with the human VKOR crystal structures available, was performed by Swiss‐Pdb viewer 4.1.0 (Spdbv) software.^38^ In particular, the superimposition of the structures of rat and mouse VKOR with the structure of human VKOR containing brodifacoum (PDB ID 6wvh) was analyzed, and a very good superimposition of the backbone of transmembrane and ER‐luminal regions was observed, reflecting the residues conservation observed in the sequence alignment. At the same way, the sidechains atoms of the binding site (i.e., residues in a neighborhood of 5 Å of brodifacoum) were very well superimposed [0.4 Å root mean square deviation (RMSD)]. The very good superimposition of the binding sites minimizes the limits of induced fit effects (that is, conformational adjustment of the binding site residues due to ligand binding), being the site already conformed to host brodifacoum, one of the reasons why the docking will be performed with this exemplary ligand.

To build the variants of the enzyme for each species, mutations suitable for docking simulations were selected from the ones presented in Tables 2 and 3, on the basis of their position with respect to the binding site. The selected residues were mutated *in silico* on the WT structure by means of the Chimera software. The selection criteria and the position of each mutated residue are deeply discussed in the Results section.

**Table 2:** Single nucleotide polymorphisms (SNPs) identified in *Rattus norvegicus* and *Rattus rattus* samples, compared to reference sequence AY423047

					*Rattus norvegicus* ‘brown rat’ (*n* = 24)			*Rattus rattus* ‘black rat’ (*n* = 35)		
Codon position	Codon and mutation	Codon and mutation	Wild‐type codon	Mutant codon	Frequency (%)	Homo	Hetero	Frequency (%)	Homo	Hetero
7^B^	Ser7Gly	S7G	AGC	GGC	0.0	0	0	2.9	0	1
12	Ala12Ala	A12A	CGG	CGA	0.0	0	0	100.0	34	1
28^B^	His28Gln	H28Q	CAC	CAG	4.2	1	0	8.6	1	2
35	Arg35Arg	R35R	CGC	CGT	0.0	0	0	2.9	0	1
36^B^	Asn36His	N36H	AAT	CAT	0.0	0	0	2.9	1	0
41	Ala41Ala	A41A	GCG	GCA	0.0	0	0	40.0	8	6
42^B^	Leu42Pro	L42P	CTC	CCC	0.0	0	0	22.9	5	3
59^A^	Trp59Arg	W59R	TGG	AGG	0.0	0	0	28.6	6	4
61^A^	Arg61Trp	R61W	CGG	TGG	16.7	2	2	0.0	0	0
82	Ile82Ile	I82I	ATA	ATT	41.7	10	0	0.0	0	0
90*	Ile90Leu	I90L	ATA	TTA	0.0	0	0	100.0	35	0
94	Leu94Leu	L94L	TTA	CTA	0.0	0	0	57.1	18	2
96^B^	Cys96Gly	C96G	TGC	GGC	4.2	0	1	2.9	0	1
107	Ile107Ile	I107I	ATC	ATA	0.0	0	0	97.1	34	0
123^B^	Ile123Phe	I123F	ATC	TTC	0.0	0	0	8.6	0	3
123^C^	Ile123Ser	I123S	ATC	AGC	4.2	1	0	0.0	0	0
137	Thr137Thr	T137T	ACC	ACT	0.0	0	0.	97.1	34	0
139^A^	Tyr139Phe	Y139F	TAT	TTT	37.5	9	0	0.0	0	0
143	Ala143Ala	A143A	GCG	GCA	0.0	0	0	85.7	17	13

*Note*: SNPs can be found in homozygosis (Homo) or heterozygosis (Hetero). Aminoacidic substitutions are indicated both in the one‐ and three‐letters codes. Frequency of mutation is intended as the total number of subjects presenting that mutation, whether in homozygosis or heterozygosis, out of the total number of subjects for that species. ^A^Mutations associated to anticoagulant rodenticide (AR) resistance; ^B^mutations newly identified in this study; ^C^mutations already known in literature, not associated with AR resistance; *the wild‐type aminoacid at position 90 is Ile in *R. norvegicus* but Leu in *R. rattus*. Silent mutations are reported in the table, not highlighted by any letter.

**Table 3:** Single nucleotide polymorphisms (SNPs) identified in *Mus musculus* samples, compared to reference sequence NM178600

					*Mus musculus* (*n* = 8)		
Codon position	Codon and mutation	Codon and mutation	Wild‐type codon	Mutant codon	Frequency (%)	Homo	Hetero
78^B^	Gln78His	Q78H	CAA	CAC	12.50	0	1
85^B^	Cys85Arg	C85R	TGC	CGC	12.50	0	1
87^B^	Phe87Leu	F87L	TTC	TTA	12.50	0	1
128^A^	Leu128Ser	L128S	TTA	TCA	50.00	1	3
139^A^	Tyr139Cys	Y139C	TAT	TGT	62.50	1	4

*Note*: SNPs can be found in homozygosis (Homo) or heterozygosis (Hetero). Aminoacidic substitutions are indicated both in the one‐ and three‐letter codes. Frequency of mutation is intended as the total number of subjects presenting that mutation, whether in homozygosis or heterozygosis, on the total number of subjects for that species. ^A^Mutations associated to anticoagulant rodenticide (AR) resistance; ^B^mutations newly identified in this study.

To prepare the enzyme structure for the docking, hydrogens were added by the Chimera software, and the catalytic cysteine residues were kept in oxidized form. To assure that the hydrogen atoms, both in the receptor binding site and in the brodifacoum, were correctly oriented, and to relax the binding site conformation to further minimize potential induced fit effects, a light energy minimization of the 5 Å neighborhood of the ligand was made in the presence of brodifacoum, roughly placed in the binding site.

Subsequently, brodifacoum was removed from the binding site and the enzyme was prepared for docking simulations, adding Kollmann partial atomic charges by means of AutoDockTools 1.5.7 (ADT) interface.^39^

Brodifacoum was firstly docked to the WT VKOR structure of house mouse, black and brown rat to check the protein structure, test the docking parameters and obtain a reference for the binding energy. Then docking simulations with the same parameters were performed for each VKOR variant of each species.

#### 2.3.3 Docking simulation parameters

Docking simulations were performed with the Autodock 4.2 software package.^39^ For the preliminary energy mapping, a grid box including the whole binding pocket (dimensions of 70 × 52 × 70 points and a spacing of 0.375 Å) was centered in the binding cavity.

For each docking simulation, the Lamarckian Genetic Algorithm was used,^40^ performing 1000 runs with 27 000 generations, an initial population of 200 individuals and, after assessing the docking convergence, with 10 × 10^6^ energy evaluations. The 1000 ligand poses resulting from the runs were structurally clustered (RMSD threshold of 1 Å).

For all docking simulations, a unique cluster was obtained, which means that the ligand conformations were all very similar, due to the tightness of the binding pocket around brodifacoum. The complex with the minimum binding energy was considered as the ‘best complex’ and used for the analysis. The structural analysis of the binding modes and interactions was performed with the software ADT, VMD 1.9.3^41^ and Spdbv.

## 3 RESULTS

### 3.1 SNPs identification

During the course of 2023, 24 brown rats, 35 black rats and eight house mice (67 total rodents) were collected and analyzed. All samples were collected in the RER in Italy (Fig. 1).

In Tables 2 and 3 SNPs that were identified in the *Vkorc1* coding sequences from our specimens are summarized, while complete details for each rodent can be found in Supporting Information Tables S1 (rats) and S2 (mice).

Looking at SNPs identified in rats, it was possible to notice several silent mutations that have been found only in the black rat, at a rather high frequency of 85.7%, 97.1% and 100%, at codon positions A143A, I107I and T137T, A12A, respectively, probably due to a different codon usage between the two species. Other silent mutations were observed with a certain frequency at codon positions: A41A, L94L in black rat, and I82I in brown rat, respectively. Moreover, all brown rats showed an Ile‐encoding WT codon 90, while black rats showed the expected Leu‐encoding WT codon 90, according to the reference A.N. XM032892517.1.

Sequence analysis showed the presence of four AR resistance‐associated missense mutations in the black rat, brown rat and house mouse, whether in homozygosis or heterozygosis (indicated by the letter A in Tables 2 and 3). They were R61W and Y139F for the brown rat, without any subject being positive for both of them. Notably, all the animals with the Y139F mutation were captured in the province of Modena (Fig. 1). For the black rat, ten subjects showed the W59R mutation, whether in homozygosis or heterozygosis, which is known to confer resistance *in vivo* in the brown rat.^42^ Regarding house mice, two missense SNPs conferring resistance to ARs^16^, ^43^ have been identified in our samples: L128S (in four mice, one being homozygous) and Y139C (in five mice, one being homozygous). Notably, three mice were heterozygous for both of these mutations, while only two subjects did not show any resistance‐associated SNP. A total of 29 out of 67 animals presented known *Vkorc1* mutations corresponding to a prevalence of 43.28%.

As well, we found the presence of newly identified mutations in all the species analyzed (indicated by the letter B in Tables 2 and 3): six in rats, prevalently in the black rat (S7G, H28Q, N36H, L42P, C96G, I123F), and three in mouse (Q78H, C85R, F87L).

Another mutation that we detected in one brown rat, I123S (indicated with a C in Table 2), had already been described in the literature despite nothing being known about its association with AR resistance.^2^, ^15^

Newly identified and known mutations underwent a structural analysis in order to assess their suitability for a molecular docking investigation to understand their possible involvement in resistance mechanisms.

### 3.2 Docking simulations

**Figure 2:**
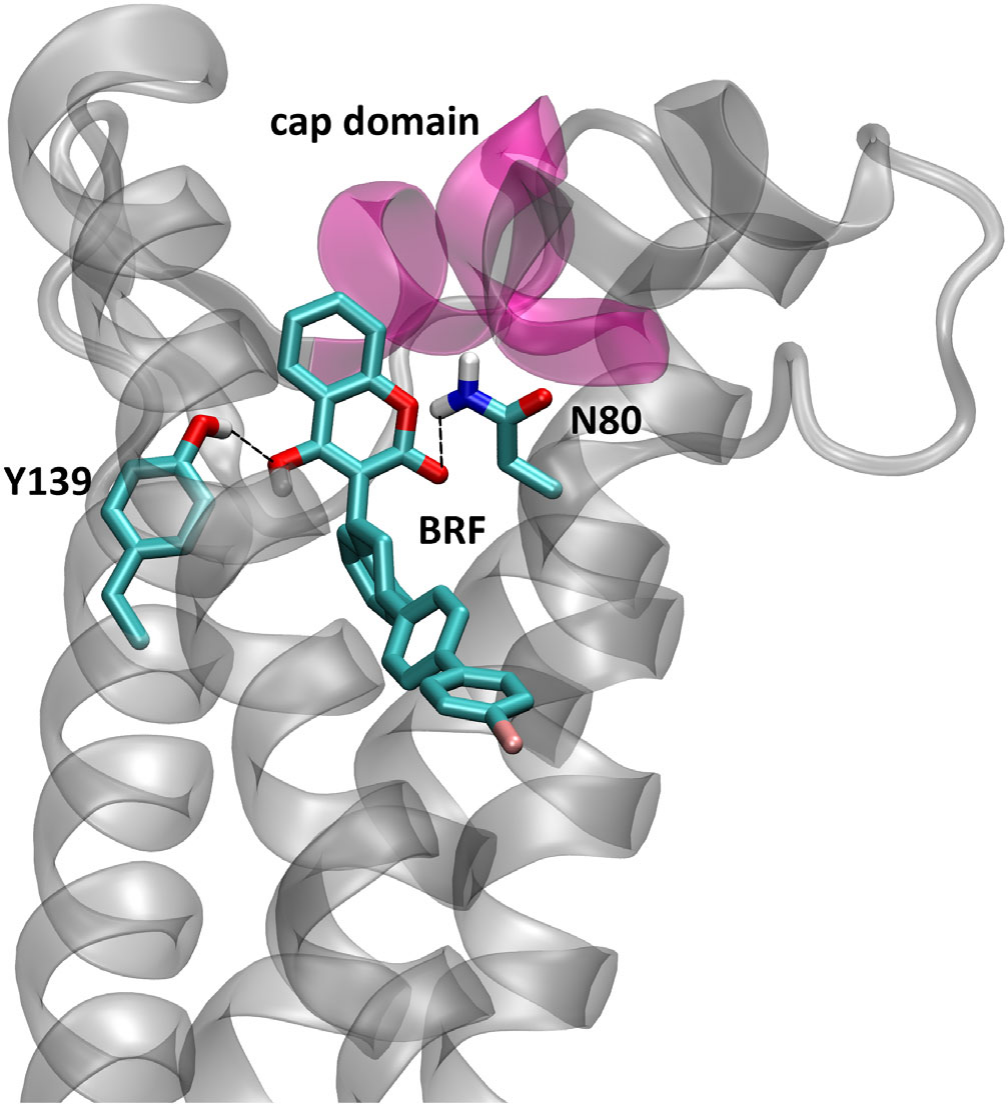
Best complex of VKOR enzyme (transparent cartoon) with brodifacoum (BRF, in sticks). Obtained by docking simulation. In pink is the cap domain. The best complexes of *Mus musculus*, *Rattus norvegicus* and *Rattus rattus* are perfectly superimposable, therefore only the former was shown here for clarity. The two key residues Y139 and N80 that form hydrogen‐bond with the substrate are highlighted in sticks. The hydrogen‐bonds are shown as dashed lines.

In the best complexes obtained from the docking simulations on WT VKOR of *M. musculus, R. norvegicus* and *R. rattus*, brodifacoum assumes the same binding mode found in the human crystal structure, preserving the known key interactions with the protein reported in the literature,^28^ that are the hydrogen‐bond formed between the Tyr139 hydroxyl and the 4‐hydroxyl group of brodifacoum, and the hydrogen‐bond formed between the amide group of Asn80 and the ketone group of brodifacoum (Fig. 2).

**Figure 3:**
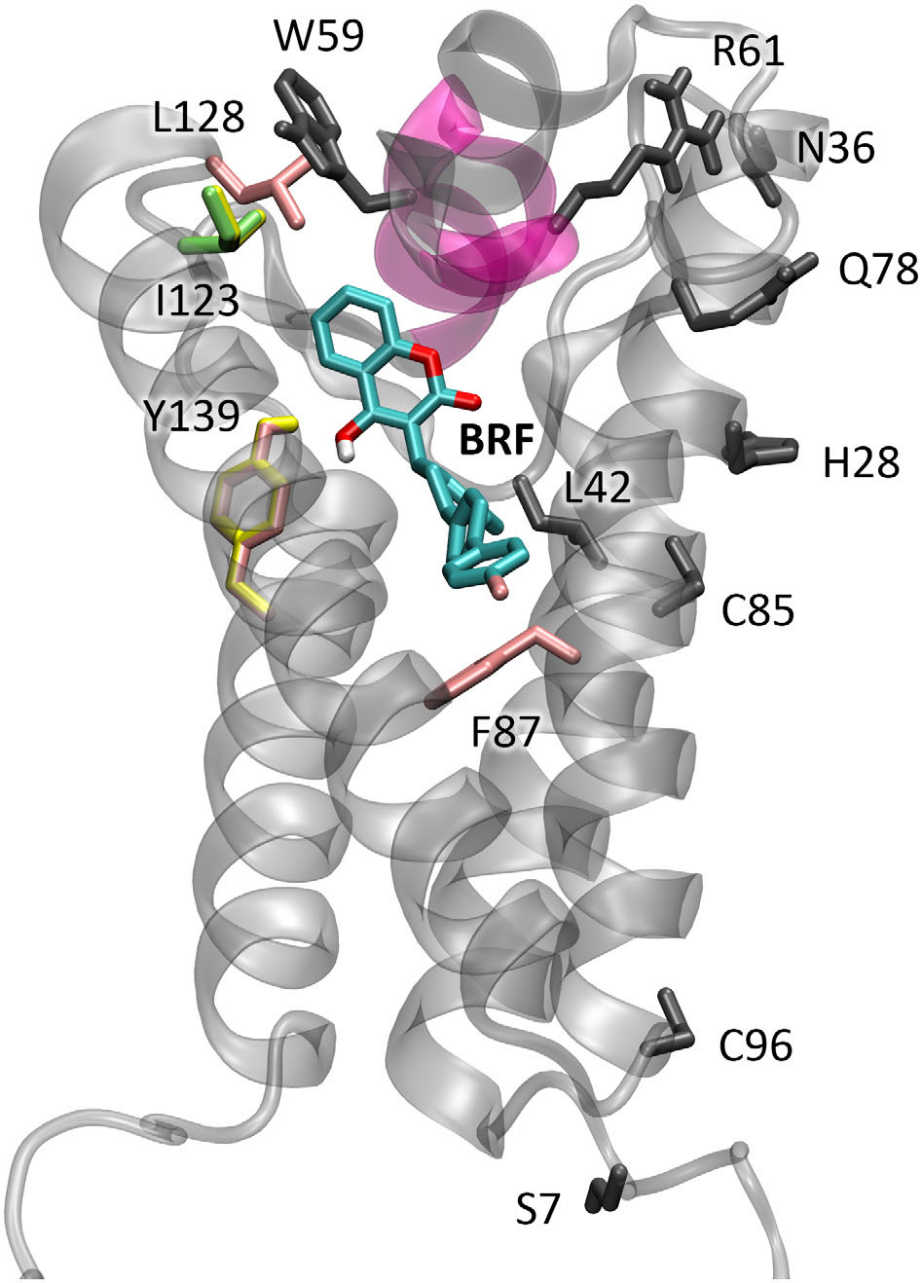
VKOR structure of *Mus musculus* (in transparent cartoon) showing all the mutations studied (in sticks). In pink is the cap domain. Mutations listed in Tables 2 and 3, known to be associated with anticoagulant resistance or found for the first time in this study are represented. In the binding site brodifacoum (BRF) is present. The colored residues represent the mutations selected for docking analysis: pink for *M. musculus*, yellow for *Rattus norvegicus*, lime for *Rattus rattus*.

To verify the effect of point mutations of VKOR protein on the binding energy of brodifacoum, several variants of the enzyme were built for each species. The mutations suitable for docking simulations were selected from the ones presented in Tables 2 and 3, on the basis of their position with respect to the binding site, therefore with the possibility to directly interfere with the binding. Figure 3 shows the position in the WT proteins structure of all the mutations, both the ones already known to be associated with anticoagulant resistance and the ones found for the first time in this study, marked respectively with A and B in Tables 2 and 3. The colored residues in Fig. 3 correspond to the mutations selected and then changed in the molecular structure to build the variants for docking simulations, a different color for different species. In Tables 4 and 5 the selection criteria and some comments on the residue features, and both their known and their putative involvement in anticoagulant resistance, are summarized.

**Table 4:** Aminoacidic substitutions due to mutations in *Vkorc1* gene found in *Rattus norvegicus* ‘brown rat’ and *Rattus rattus* ‘black rat’

*Rattus norvegicus* ‘brown rat’/*Rattus rattus* ‘black rat’				
Mutation	Position	Features	Suitable for docking	Confers AR resistance
S7G	Near the cytoplasmic interface	Probably not affecting the binding affinity	No	NA
H28Q	In the ER‐luminal region; exposed to the solvent	The mutation that is known to confer resistance in rat is H28A.^28^ However, H28Q is known to confer resistance in human,^44^ therefore it is likely to have the same behavior in rat. In fact, His28 stabilizes the loop 1 in the ER‐luminal region by a hydrogen‐bond with Tyr39, therefore its mutation could destabilize the cap domain.	No	To warfarin, in human^44^
N36H	In the ER‐luminal region, exposed to the solvent	It could destabilize the cap domain	No	NA
L42P	Close to the catalytic Cys43; exposed to the solvent	Most likely the rigid proline induces a deformation in the structure; being close to the catalytic site, this could affect the catalytic cycle	No	NA
W59R	In the middle of the cap domain; exposed to the solvent	It reverses side chain polarity; it could destabilize the cap domain	No	Yes (*in vivo*)^42^
R61W	In the middle of the cap domain; exposed to the solvent	It reverses side chain polarity; it could destabilize the cap domain	No	Yes^45^
C96G	Near the cytoplasmic interface	Probably not affecting the binding affinity	No	NA
I123F I123S	Close to the ER‐luminal region, but in contact with the substrate	I123S was reported as detected in Italian rats, but not known if associated to AR resistance.^15^ As for L128S in mouse, it reverses side chain polarity inserting a polar side chain (serine) in a hydrophobic region, with hydrophobic contacts with the substrate. Both Ile123 mutations are suitable for docking	Yes	A similar mutation (I123N) is known for resistance in humans^46^
Y139F	Close to the ER‐luminal region, but in contact with the substrate	One of the two key residues that form hydrogen‐bonds with the substrate; close to the catalytic site	Yes	Yes (warfarin)^2^, ^9^

*Note*: Their position in vitamin K epoxide reductase (VKOR) protein is described and some features of each mutation are discussed. AR, anticoagulant rodenticide; ER, endoplasmic reticulum; NA, no information about the impact of that mutation on the resistance to AR was available in the literature.

**Table 5:** Aminoacidic substitutions due to mutations in *Vkorc1* gene found in *Mus musculus* ‘house mouse’

*Mus musculus*				
Mutation	Position	Features	Suitable for docking	Confers AR resistance
Q78H	In the anchor region of the cap domain; exposed to the solvent	It could destabilize the cap domain	No	NA
C85R	Exposed to membrane lipidic environment; not in contact with the substrate	Single cysteine residue	No	NA
F87L	In contact with substrate	Phe ring in stacking with side group of brodifacoum: such interaction is relevant to enhance the inhibition by the substrate	Yes	NA
L128S	Close to the ER‐luminal region, but in contact with the substrate	It reverses side chain polarity inserting a polar side chain (serine) in a hydrophobic region, with hydrophobic contacts with the substrate	Yes	Yes^2^, ^28^
Y139C	Close to the ER‐luminal region, but in contact with the substrate	One of the two key residues that form hydrogen‐bonds with the substrate; close to the catalytic site	Yes	Yes^16^, ^43^
L128S/Y139C	As described earlier	Double mutation found in one mouse in double heterozygosis	Yes	Yes^47^

*Note*: Their position in vitamin K epoxide reductase (VKOR) protein is described and some features of each mutation are discussed. AR, anticoagulant rodenticide; ER, endoplasmic reticulum; NA, no information about the impact of that mutation on the resistance to AR was available in the literature.

The docking simulations on the VKOR variants show the ligand in the same orientation as in the WT proteins. However, in almost all the variants, some critical interactions were missed, causing a more unfavorable binding energy with respect to the WT complex. In Table 6 the binding energy of the best complex obtained from the docking of brodifacoum with each variant is shown. To assess if the differences in binding energy between the WT and mutated complexes are significant, the binding energy distributions of each docking were analyzed and they appear well separated (Fig. S2).

**Table 6:** Minimum binding energy (kcal/mol) of the complex of vitamin K epoxide reductase (VKOR) enzyme with brodifacoum for all the variants of all species, obtained by the docking simulations

*Mus musculus*		*Rattus norvegicus*		*Rattus rattus*	
Wild‐type (WT)	−15.06	WT	−15.33	WT	−15.22
F87L	−14.61	I123S	−14.92	I123S	−14.83
L128S	−14.90	Y139F	−14.52	I123F	−15.39
Y139C	−14.37				
L128S/Y139C	−14.13				

**Figure 4:**
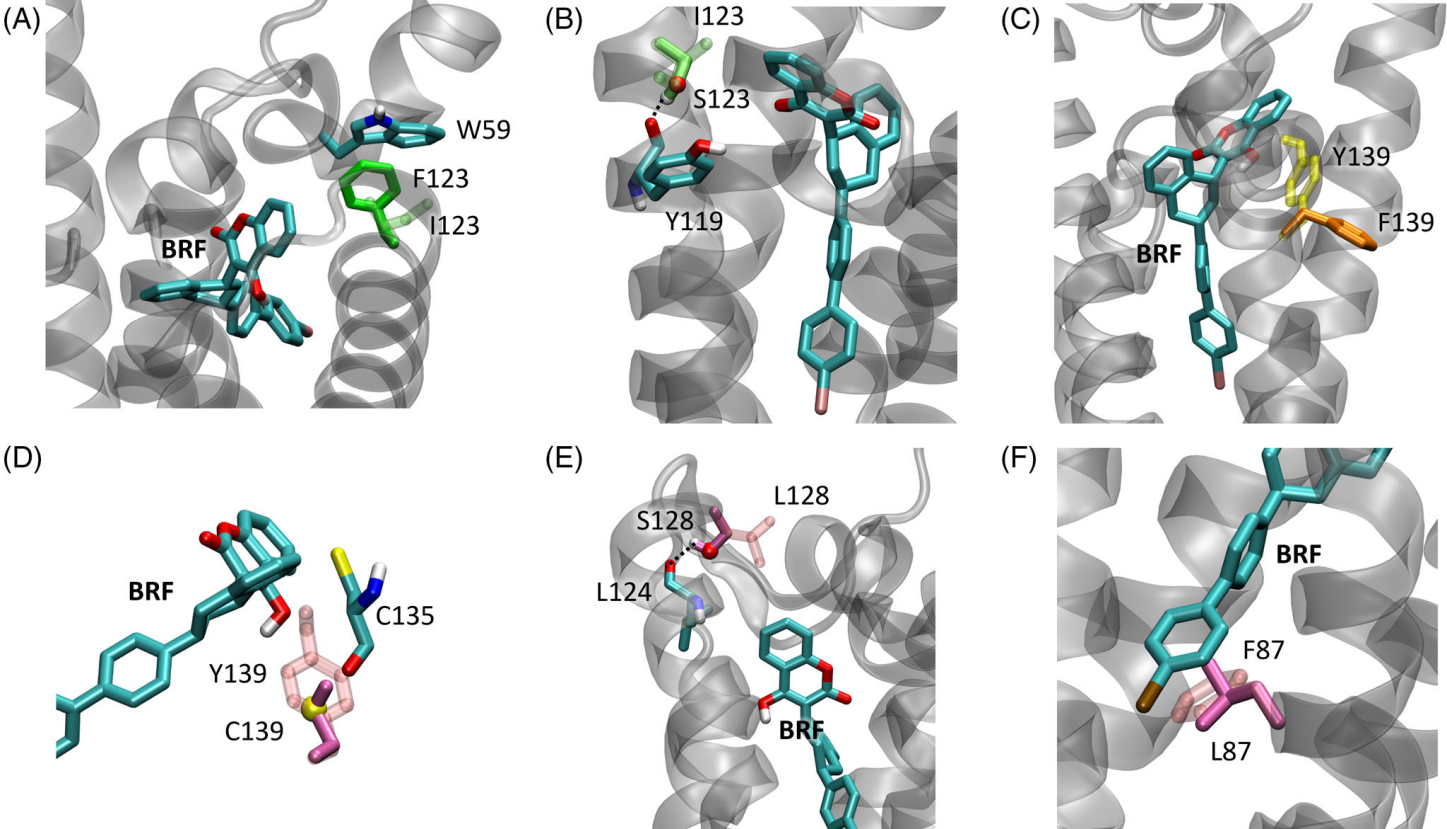
Docked complexes of the selected variants of VKOR (transparent cartoon) with brodifacoum (BRF). For each variant, the WT residue is superimposed in transparent, while the mutated one is opaque in a darker color. On the latter, polar atoms are colored by type (A) I123F mutation. The stacking with W59 is highlighted. (B) I123S mutation. The hydrogen‐bond of S123 sidechain oxygen with Y119 backbone oxygen is highlighted with a dotted line. (C) Y139F mutation. (D) Y139C mutation. The proximity of C135 residue is highlighted. (E) L128S mutation. The hydrogen‐bond of S128 sidechain oxygen with L124 backbone oxygen is highlighted with a dotted line. (F) F87L mutation.

As mentioned earlier, all the best complexes of the VKOR variants show – to a different extent – a higher binding energy (less favorable) than the WT complex. The only exception is I123F mutation of the black rat. In this case, in fact, the mutation of isoleucine to phenylalanine enhances the hydrophobic contacts with brodifacoum ring and, in addition, allows an aromatic ring stacking with the side chain of Trp59, favoring the mutation (Fig. 4(A)). Therefore, this mutation probably does not have relevant effects on anticoagulant resistance.

For both rat species, also the I123S mutation was tested, giving a binding energy only slightly higher than WT proteins. This mutation should bring a polar side chain in contact with the hydrophobic ring of brodifacoum, changing the ligand environment. However, not only the mutated serine is smaller than the native isoleucine, but serine finds a favorable rotamer moving its hydroxyl group away from the side group of the substrate. In this way, its sidechain forms a hydrogen bond with the backbone oxygen of Tyr119, and exposes its apolar beta carbon towards the brodifacoum ring (Fig. 4(B)). Therefore, the interaction with the ligand is only partially affected.

The Y139F mutation in the brown rat is one of the more common mutations found in Europe that leads to anticoagulant resistance.^2^ The binding energy of this variant is, in fact, clearly higher than the WT protein, indicating an unfavorable binding of AR. The tyrosine is replaced with a residue – phenylalanine – that, even if with the same bulkiness due to the aromatic ring, has lost the polarity and, above all, the ability to form the key hydrogen‐bond with the ligand hydroxyl. In addition, the less favorable interaction is pointed out by the external bending of the phenylalanine side chain obtained with the modeling of the mutated residue, whose rotamer faces away from the substrate (Fig. 4(C)).

Regarding the mouse variants, more mutations were analyzed with docking simulations than in the rat, due to their localization in the binding site. The Y139C mutation, analogously to Y139F, seems to have a high effect on the binding energy, in agreement with the literature,^2^, ^48^ due again to the disruption of the hydrogen‐bond of Tyr139 hydroxyl. In this case the cysteine residue that replaces tyrosine forms a new hydrogen‐bond with the backbone oxygen of the neighborhood Cys135, not being able to reach the 4‐hydroxyl group of brodifacoum (Fig. 4(D)) as native Tyr139 does. Interestingly, the proximity of the mutated Cys139 with the catalytic Cys135 leads to the question of whether an oxidization event could occur between the two close cysteines, interfering with the catalytic cycle.

Contrarily to what is expected from the resistance behavior reported in the literature,^2^, ^28^ the docking analysis on the L128S mutation shows the smallest increase in binding energy, as it happens for the similar mutation I123S in rat. The mutated Ser128 in mouse shows, in fact, an analogous behavior of Ser123 in rat, turning away its hydroxyl group from brodifacoum to form an advantageous hydrogen‐bond between its hydroxyl group and the backbone oxygen of Leu124, and exposing side chain beta carbon to the substrate (Fig. 4(E)).

In the double mutant L128S/Y139C the effects of both mutations are enhanced, bringing the binding energy to the highest value, therefore indicating a possible very high resistance to ARs.

The F87L mutation shows again an increase in the binding energy than the WT complex, due to the loss of the stacking of the phenylalanine ring with the ring of brodifacoum (Fig. 4(F)). This kind of interaction is, in fact, reported to be relevant to enhance the inhibition by the substrate,^28^ and its loss indicates a less favorable binding to AR.

## 4 DISCUSSION

AR resistance represents a public health and environmental issue, due to the increased spread of zoonotic pathogens^3^ and to the unwanted exposure of non‐target species,^22^, ^23^, ^24^, ^25^ and this is mainly caused by the insurgence of mutations in *Vkorc1*.^20^, ^42^ The presence of mutations of this gene in local rodent populations has been increasingly studied worldwide in the last decade^9^, ^10^, ^11^, ^14^, ^49^ however, only a small amount of published data is available regarding Italian rodents,^15^, ^16^, ^18^ with no proof of AR resistance‐associated mutations in rats and just one mouse found positive for Y139C in the Pontine Archipelago.^16^ However, according to the recent maps available on the Rodenticide Resistance Action Committee (RRAC) website (https://guide.rrac.info/resistance-maps/norway-rat/europe/italy.html), a few cases of Y139 mutations have been detected in the brown rat in Italy after 2019 (one case of Y139F in northern Italy and six cases of Y139S in southern Italy), but we have found no published data supporting this information.

In the present study, we focused on the presence of *Vkorc1* SNPs in rodents from RER, in Italy. Nine brown rat samples were found to be mutated in homozygosis for Y139F, a missense mutation that is well‐known as one of the most diffused in western Europe, conferring resistance to both FGARs and SGARs,^2^, ^9^ behavior that is consistent with the results we obtained by molecular docking simulations. Moreover, another four brown rats showed R61W missense mutation, two in homozygosis and two in heterozygosis, which was identified so far in the black rat as associated to warfarin resistance.^45^ However, no brown rat in the present study resulted positive for both mutations. In addition, ten black rats showed the presence of the W59R substitution (six of them in homozygosis): this mutation was identified in brown rats from Argentina, and associated to AR resistance through *in vivo* tests, even though it strongly reduces the basal activity of the VKOR enzyme, thus exposing the animals to an increased risk of internal bleeding even in the absence of ARs.^42^

Regarding house mice, despite the small number of samples examined (*n* = 8), the presence of L128S and Y139C SNPs, the last prevailing in north‐eastern Europe and conferring a strong resistance to FGARs, bromadiolone and less severely to difenacoum,^16^, ^43^ is noteworthy. Both mutations have been detected prevalently in heterozygosis with a frequency from 50% to 60% respectively, while two animals resulted being in homozygosis for one or the other of the two SNPs, confirming a diffuse presence of these non‐synonymous SNPs in the house mice in Italy^16^ and, as recently reported, in other Mediterranean countries, like Lebanon.^13^ Our docking simulations results have shown how – as expected – the presence of both mutations make the binding with AR even less favorable than single mutation, mutually enhancing their effect.

These results represent the first evidence of AR‐resistance mutations in rodents from RER and, to the best of our knowledge, the first published data assessing resistance in Italian rats.

Regarding silent mutations, it is interesting to note that two of the most common ones, I107I and T137T, that we both found in homozygous form in 34/35 black rats (97.1%), have already been reported in the same species from New Zealand,^50^ India^51^ and recently in Singapore,^11^ as well as being reported in brown rats from Indonesia and Thailand.^42^ The occurrence of these silent mutations in both rat species is in agreement with the historical origin of the brown rat from south‐western China about 1.3 million years ago^52^ and of the black rat from South Asia and Indochina regions.^53^

We also found several mutations that were never described before; molecular docking simulations, predicting the binding mode and the binding energy of the complex, helping us to evaluate, for the suitable ones, the interaction changes and the possible role on resistance.

Looking at the binding energy behavior of all variants complexed with brodifacoum, the mutations selected for docking, with the exception of I123F, appear to play a role, to a different extent, in resistance to brodifacoum. All the mutations involving Tyr139, whose hydrogen‐bond with substrate is crucial for binding, are strong candidates to express AR resistance. In addition, Tyr139 is close to the catalytic Cys132‐Cys135 site, therefore also a structural interference with the catalytic cycle is possible. Unexpectedly, also F87L, while maintaining the residue bulkiness and hydrophobicity, disrupts the stacking interaction responsible for the strong inhibition by the substrate. Interestingly, the other analyzed mutations face to the upper region of the VKOR binding pocket, and therefore can be related to resistance both to the FGARs and to the SGARs, whose binding mode occupy this region of the pocket. Instead, the F87L mutation is placed in the lower part of the binding pocket that only the large side group of SGARs (like brodifacoum) can reach, suggesting the conferring of a resistance to this second group of ARs.

Regarding the I123S mutation, it has been identified in one sample of *R. norvegicus*: this substitution was already found in some animals of the same species in a rural Venetian population in Italy,^15^ suggesting a probable local evolution of this mutation. Even if it is not experimentally known whether I123S exerts a role in AR resistance in brown rat, it is located in the same position as I123N, a SNP known to confer AR resistance in humans.^46^ Both mutated amino acids – asparagine and serine – share a polar and hydrophilic character, unlike the hydrophobic WT amino acid isoleucine, suggesting a similar involvement in resistance^15^; nevertheless, serine has a smaller side chain that perturbs little the ligand environment, showing, from the docking analysis, a binding more favorable than expected (and probably than asparagine), even if less favorable than the WT residue.

About the other mutations already reported in the literature, but not suitable for docking, it is worth mentioning the finding of the H28Q substitution in both of the rat species. This mutation has not been identified before in the three species analyzed, but it is known to cause warfarin resistance in humans.^44^ Due to the fact that His28 stabilizes the loop 1 in the ER‐luminal region by a hydrogen‐bond with Tyr39, its mutation probably destabilizes the cap domain, and it is reliable that it acts in the same way both in human and in rat.

Other newly described mutations, not suitable for docking, could be involved in resistance due to their position in or near critical areas of the protein, such as the cap domain (N36H for *Rattus* spp. and Q78H for *M. musculus*) or the catalytic site (L42P, *Rattus* spp.). However, this remains a hypothesis: other approaches, such as molecular dynamics simulations, could help to evaluate the role on resistance of mutations not suitable for docking, and should be considered for further studies.

## 5 CONCLUSIONS

This study has shown, for the first time, the presence of AR resistance‐associated mutations of the *Vkorc1* gene in rodents from RER, and in rats from Italy. Sequencing of the complete *Vkorc1* gene has allowed us to identify new mutations, outside from the more studied third exon, that could have a role in conferring resistance to ARs. Docking analysis has proven a useful tool to evaluate the possible impact of mutations on resistance, at least for those amino acids exposed in the binding pocket.

These data could be useful to drive more effective rodent pest management policies, and should be expanded to gather more data from the rest of Italy: in fact, the absence of an AR reference laboratory, at least in Italy, hampers the monitoring of the overall quality and effectiveness of rodent pest management measures, despite them being frequent in urban, industrial and zootechnic areas. Moreover, this limits the awareness about the need of risk mitigation measures by pest control operators.

In this scenario, it is therefore urgent to adopt effective practices of resistance management and risk mitigation, strictly avoiding the use of ARs as routine practice not well supported by monitoring. Among the preventive alternative methods, rat proofing, the use of traps, the alternating use of ARs with cholecalciferol‐based products recently reintroduced also in the Italian market, the monitoring and continuous maintenance of sites at risk, the correct management of unconsumed baits and dead rodents, represent overall effective means included in the integrated pest management strategy.

The application of an integrated control strategy represents a concrete response to the risk of a progressive decline in the effectiveness of ARs, which effectiveness has to be preserved as an indispensable tool where the rodent presence can threaten human and animal health.

The discovery of AR resistance SNPs in the 43.28% of tested rodents in the present study sounds as an alarm bell that, together with the mitigation of risks to non‐targets, has to be considered a stimulus to strengthen cooperation between research bodies, trade associations and rodenticide distributors.

## AUTHORS CONTRIBUTIONS

Conceptualization: MD, RB, EC and AR; resources: EC, RV, RB, CV, FMD, RG, GP and MD; methodology: EC, AR and EP; investigation: AR, GR and EC; formal analysis: AR and EP; software: AR; data curation: AR and EC; visualization: AR, EP and EC; writing – original draft: AR, EP and EC; writing – review and editing: GR, RV, RB, FMD, RG, CV, GP and MD; supervision: EC and MD; funding acquisition: AR.

## CONFLICT OF INTEREST STATEMENT

The authors declare no conflicts of interest.

## Supporting information


**Figure S1.** Clustal Omega multiple sequence alignment of human, *Mus musculus, Rattus norvegicus*, and *Rattus rattus* VKOR protein.
**Figure S2.** Binding energy distributions of all the 1000 complexes obtained from the docking of each variant. Each distribution is well separate, assuring that the differences in binding energy between the complexes are significant.

**Table S1.** Detail of mutations found in every rat analyzed, with species information and death cause/location (if available). Capital mutations indicate single nucleotide polymorphisms (SNPs) at a DNA level, the following lowercase mutation indicates the aminoacidic substitution. 0: no mutation; 0/1: heterozygous mutation; 1: homozygous mutation.

**Table S2.** Detail of mutations found in every mouse analyzed, with species information and death cause/location (if available). Capital mutations indicate single nucleotide polymorphisms (SNPs) at a DNA level, the following lowercase mutation indicates the aminoacidic substitution. 0: no mutation; 0/1: heterozygous mutation; 1: homozygous mutation.

## Data Availability

The data that supports the findings of this study are available in the supplementary material of this article.
