# Supplementary material for: New polymorphisms of Vkork1 gene related to anticoagulant resistance of rats and mice in Italy
Source: Pest Manag Sci. 2025 Jan 15;81(6):2869–80. doi: 10.1002/ps.8652 (PMC12074628; doi:10.1002/ps.8652)
Supplement: Supplementary file 1 — Figure S1. Clustal Omega multiple sequence alignment of human, Mus musculus, Rattus norvegicus, and Rattus rattus VKOR protein. Figure S2. Binding energy distributions of all the 1000 complexes obtained from the docking of each variant. Each distribution is well separate, assuring that the differences in binding energy between the complexes are significant. [file PS-81-2869-s002.pptx]

## Slide 1
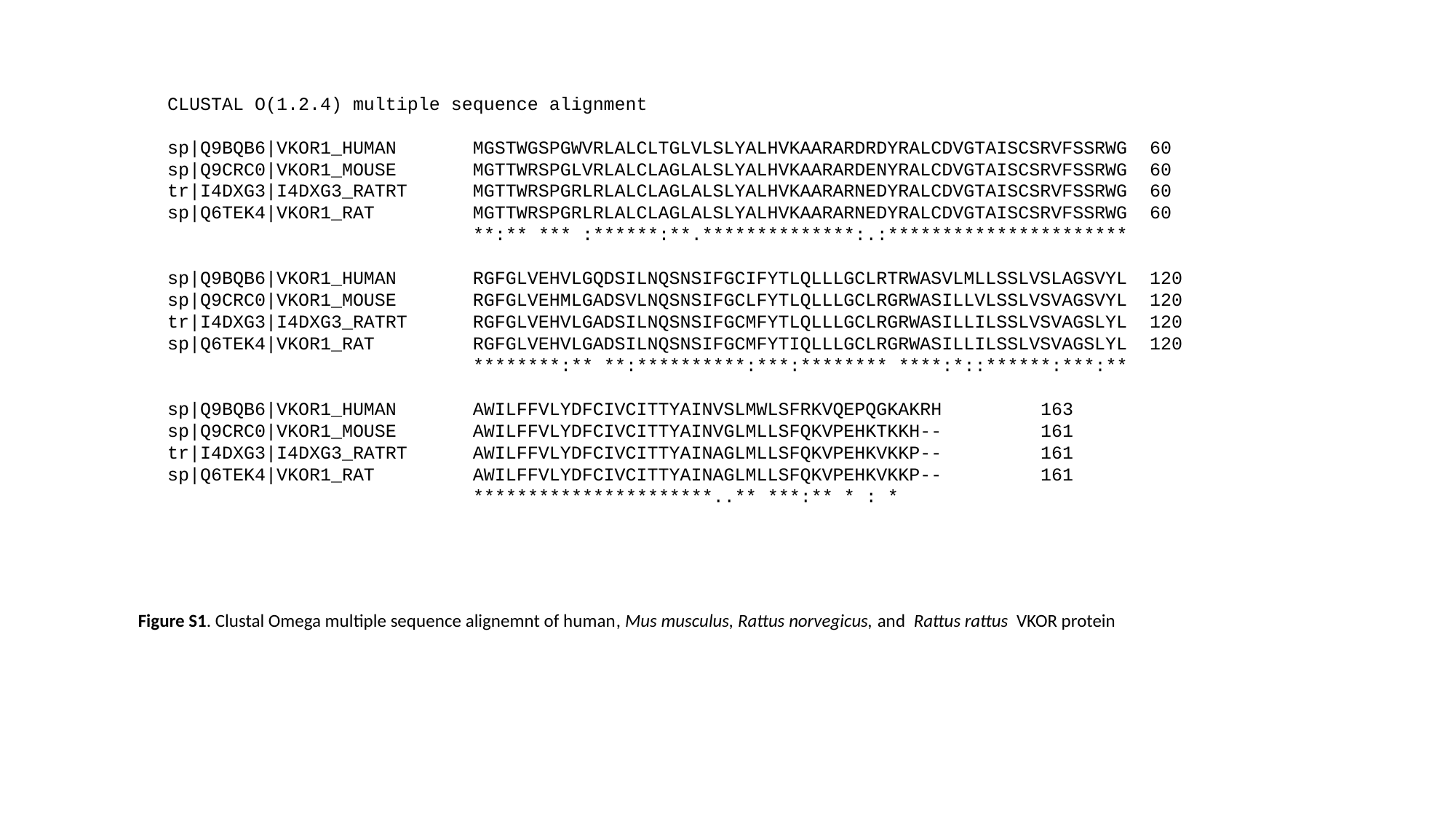

CLUSTAL O(1.2.4) multiple sequence alignment
sp|Q9BQB6|VKOR1_HUMAN MGSTWGSPGWVRLALCLTGLVLSLYALHVKAARARDRDYRALCDVGTAISCSRVFSSRWG	60
sp|Q9CRC0|VKOR1_MOUSE MGTTWRSPGLVRLALCLAGLALSLYALHVKAARARDENYRALCDVGTAISCSRVFSSRWG	60
tr|I4DXG3|I4DXG3_RATRT MGTTWRSPGRLRLALCLAGLALSLYALHVKAARARNEDYRALCDVGTAISCSRVFSSRWG	60
sp|Q6TEK4|VKOR1_RAT MGTTWRSPGRLRLALCLAGLALSLYALHVKAARARNEDYRALCDVGTAISCSRVFSSRWG	60
 **:** *** :******:**.**************:.:**********************
sp|Q9BQB6|VKOR1_HUMAN RGFGLVEHVLGQDSILNQSNSIFGCIFYTLQLLLGCLRTRWASVLMLLSSLVSLAGSVYL	120
sp|Q9CRC0|VKOR1_MOUSE RGFGLVEHMLGADSVLNQSNSIFGCLFYTLQLLLGCLRGRWASILLVLSSLVSVAGSVYL	120
tr|I4DXG3|I4DXG3_RATRT RGFGLVEHVLGADSILNQSNSIFGCMFYTLQLLLGCLRGRWASILLILSSLVSVAGSLYL	120
sp|Q6TEK4|VKOR1_RAT RGFGLVEHVLGADSILNQSNSIFGCMFYTIQLLLGCLRGRWASILLILSSLVSVAGSLYL	120
 ********:** **:**********:***:******** ****:*::******:***:**
sp|Q9BQB6|VKOR1_HUMAN AWILFFVLYDFCIVCITTYAINVSLMWLSFRKVQEPQGKAKRH	163
sp|Q9CRC0|VKOR1_MOUSE AWILFFVLYDFCIVCITTYAINVGLMLLSFQKVPEHKTKKH--	161
tr|I4DXG3|I4DXG3_RATRT AWILFFVLYDFCIVCITTYAINAGLMLLSFQKVPEHKVKKP--	161
sp|Q6TEK4|VKOR1_RAT AWILFFVLYDFCIVCITTYAINAGLMLLSFQKVPEHKVKKP--	161
 **********************..** ***:** * : *
Figure S1. Clustal Omega multiple sequence alignemnt of human, Mus musculus, Rattus norvegicus, and Rattus rattus VKOR protein

## Slide 2
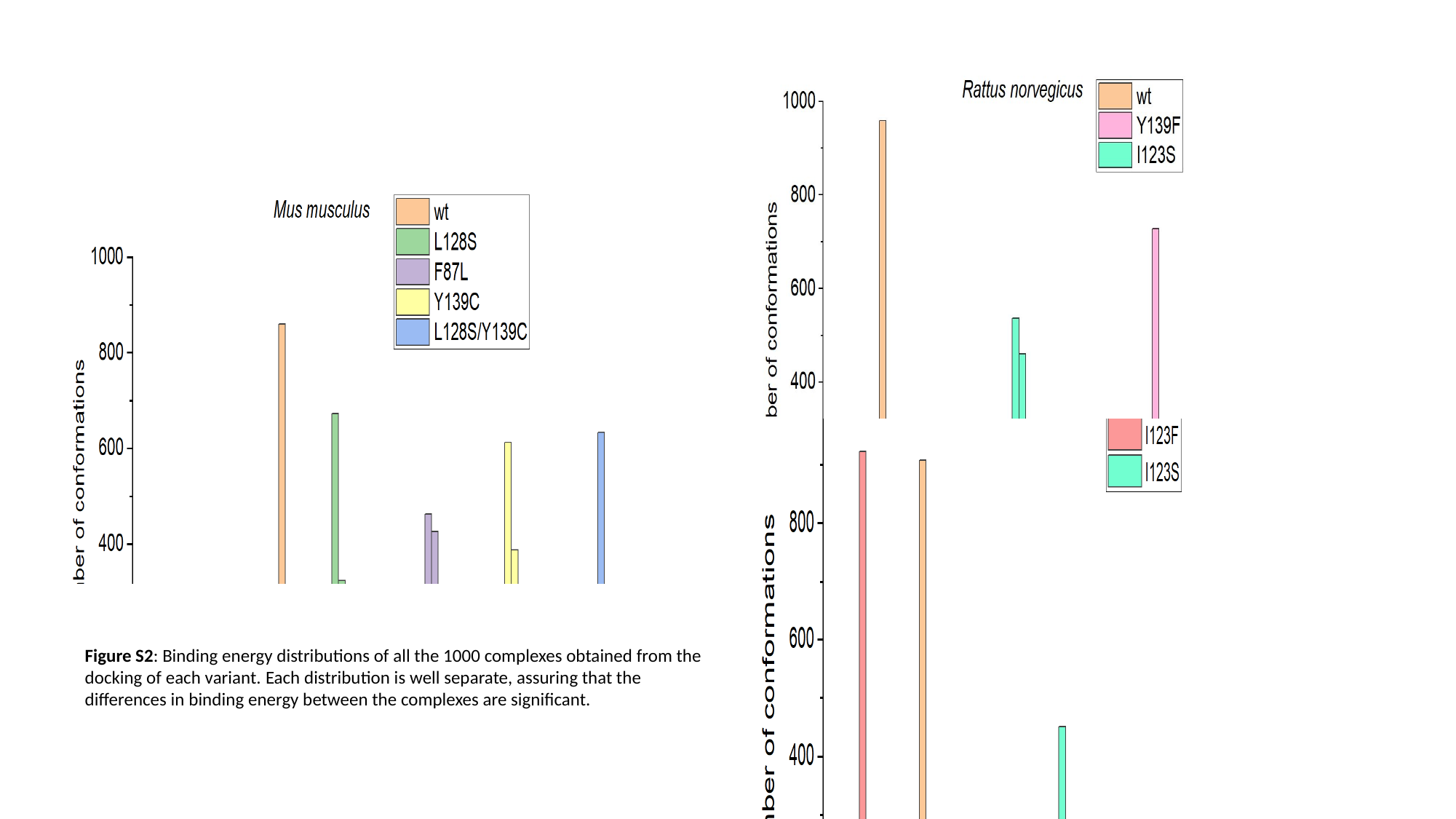

Figure S2: Binding energy distributions of all the 1000 complexes obtained from the docking of each variant. Each distribution is well separate, assuring that the differences in binding energy between the complexes are significant.
